# History of the Medical Examining Board of Georgia

**Published:** 1898-11

**Authors:** E. R. Anthony

**Affiliations:** Griffin, Ga., Secretary of the Board


### HISTORY OF THE MEDICAL EXAMINING BOARD OF GEORGIA.

BY E. R. ANTHONY, M.D., GRIFFIN, GA.,

Secretary of the Board.

To elevate the standard of medical education in the United States has been, for the past thirty years, the great aim of the leading men of the medical profession in every State in the Union. Actuated by an intense devotion to science, and a high sense of duty to the public, they have worked faithfully to devise ways and means to this end, and to make the doctorate conferred by our medical colleges worthy of the respect that should be accorded it by the members of the other learned professions and by men of education generally. Logical essays and eloquent orations from various members of different medical societies, whenever and wherever convened, failed for years and years to point out the remedy for the growing evil of graduating from our medical colleges illiterate and incompetent men.

The medical journals, in editorials of no uncertain meaning, condemned and opposed this prostitution of a great educational trust, but suggested no remedy. Finally, like a happy inspiration, the idea came to some one to suggest State Medical Examining Boards. The leading countries of Europe, Germany, Austria, England, and others had Examining Boards, why not our States? This seemed to be the long sought-for remedy, and the rank and file of our profession in every State of the Union began to demand laws of their State legislatures that would create Examining Boards, and advance the cause of higher medical education. Twenty-six States have succeeded in having such laws passed, but in many instances only after a bitter struggle.

It is with feelings of pardonable pride that we can point to Georgia's name on this "Roll of Honor." It gives me pleasure to speak of the great aid given this movement by the medical journals and the medical colleges in our State. Their assistance, in bringing about these sweeping reforms, recently instituted in our

State, have been invaluable. It gives us greater faith in the higher destiny of our profession to know that every physician, of any prominence in Georgia, hopefully and persistently worked for the passage of this law. While this is true, I should fail in justice and in courtesy alike to several worthy gentlemen, did I not speak more particularly of their efforts in behalf of the enactment of this law. To the untiring energy of many leading members of the profession in Georgia, the unselfish interest of Mr. Fleming, and the masterly manner in which the bill was managed by Mr. Fouché, is largely due the passage of the bill by the legislature of 1894. While the present law may not be entirely satisfactory to some, in my opinion, it is one of the best that has been adopted by any of the twenty-six States that have Examining Boards. Has the execution of this law by the gentlemen appointed for this purpose by the governor been satisfactory? We believe so.

It has been the endeavor of the members of the board, in the performance of their duties, to be governed by justice and moderation. Conservatism and charity, in the enforcement of any new law, ever appeals to the better sense of man. It takes time to bring about any great advance in religious, political or scientific reforms.

Hence, the board adopted for its guidance the old Latin maxim, *festina lente.* We have made haste slowly, but we feel now that the experimental age or stage of this law has passed, and that we would fall short of our duty did we not make our examinations more rigid, and grade the answers to questions more strictly. It has been a question with the board whether, under the law, we had the right, in grading the papers of some applicants, to take into consideration the evidence of their not having the essential elements of a good English education.

We have licensed a good many when it was painfully evident that they did not possess this qualification. We were apprehensive that we might overstep our authority if we allowed this question to influence us when passing upon their proficiency in the medical branches, and besides, beyond doubt, this is a question to be regulated by the medical colleges, which should have certain educational requirements for matriculation.

The first meeting of the board was held in Atlanta, January

21st, 1895, the following gentlemen comprising the board: Dr. A. A. Smith, Dr. James B. Baird; Dr. F. M. Ridley; Dr. W. A. O'Daniel and Dr. E. R. Anthony. Dr. F. M. Ridley was elected president and immediately resigned. Dr. A. A. Smith was then elected president and Dr. F. M. Ridley secretary. In 1896 Dr. E. R. Anthony was elected president and Dr. W. A. O'Daniel secretary. In 1897 Dr. F. M. Ridley was elected president and Dr. O'Daniel secretary. In 1896 Dr. J. B. Baird resigned as a member of the board to accept a chair in the Southern Medical College, and Dr. J. C. Olmsted was appointed by the governor to fill the vacancy. In 1897 Dr. Olmsted resigned to accept a chair in the Southern Medical College, and Dr. J. B. S. Holmes was appointed to fill the vacancy. There has been no change in the *personnel* of the board since then. The officers of the board at present are J. B. S. Holmes, M.D., president, and Dr. E. R. Anthony, secretary. Since the organization of the board eighteen sessions have been held and 382 applicants have been licensed to practice, as follows: In 1895, 107; in 1896, 62; in 1897, 106; in 1898, 107. About ten per cent. of all applicants have been rejected. In future the proceedings of the sessions of the board, and the results of the examinations, giving the number of applicants from each medical college examined, the number licensed and the number rejected and the percentage made, will be published in THE ATLANTA MEDICAL AND SURGICAL JOURNAL. This plan has been adopted by the boards of several States, and we believe it will prove beneficial. The creation of examining boards in so many States has compelled the medical colleges to elevate their standard for graduation and has forced them to exercise more care in the granting of diplomas. Another good effect of this law is that it deters a large proportion of the incompetent from selecting the profession of medicine as a life avocation. Without any desire to reflect upon the proficiency of the earlier classes licensed by the board, we must say it has been a source of great pleasure to the members of the board to observe the improvement each year has shown in the preparation and fitness of the applicants.

